# Changes in Selected Biochemical Indices Resulting from Various Pre-sampling Handling Techniques in Broilers

**DOI:** 10.1186/1751-0147-53-31

**Published:** 2011-05-13

**Authors:** Petr Chloupek, Iveta Bedanova, Jan Chloupek, Vladimir Vecerek

**Affiliations:** 1 Department of Veterinary Public Health and Toxicology, Faculty of Veterinary Hygiene and Ecology, University of Veterinary and Pharmaceutical Science Brno 612 42 Brno, Czech Republic; 2 Department of Nutrition, Livestock Breeding and Animal Hygiene, Faculty of Veterinary Hygiene and Ecology, University of Veterinary and Pharmaceutical Sciences in Brno, 612 42 Brno, Czech Republic

## Abstract

**Background:**

Since it is not yet clear whether it is possible to satisfactorily avoid sampling-induced stress interference in poultry, more studies on the pattern of physiological response and detailed quantification of stress connected with the first few minutes of capture and pre-sampling handling in poultry are required. This study focused on detection of changes in the corticosterone level and concentrations of other selected biochemical parameters in broilers handled in two different manners during blood sampling (involving catching, carrying, restraint, and blood collection itself) that lasted for various time periods within the interval 30-180 seconds.

**Methods:**

Stress effects of pre-sampling handling were studied in a group (n = 144) of unsexed ROSS 308 broiler chickens aged 42 d. Handling (catching, carrying, restraint, and blood sampling itself) was carried out in a gentle (caught, held and carried carefully in an upright position) or rough (caught by the leg, held and carried with lack of care in inverted position) manner and lasted for 30 s, 60 s, 90 s, 120 s, 150 s, and 180 s. Plasma corticosterone, albumin, glucose, cholesterol, lactate, triglycerides and total protein were measured in order to assess the stress-induced changes to these biochemical indices following handling in the first few minutes of capture.

**Results:**

Pre-sampling handling in a rough manner resulted in considerably higher plasma concentrations of all biochemical indices monitored when compared with gentle handling. Concentrations of plasma corticosterone after 150 and 180 s of handling were considerably higher (P < 0.01) than concentrations after 30-120 s of handling regardless of handling technique. Concentrations of plasma lactate were also increased by prolonged handling duration. Handling for 90-180 seconds resulted in a highly significant elevation of lactate concentration in comparison with 30 s handling regardless of handling technique. Similarly to corticosterone concentrations, a strong positive correlation was found between plasma lactate and duration of pre-sampling handling. Other biochemical indices monitored did not show any correlation pattern in connection with duration of pre-sampling handling.

**Conclusions:**

These results indicate that the pre-sampling procedure may be a considerably stressful procedure for broilers, particularly when carried out with lack of care and exceeding 120 seconds.

## Background

In the last few decades, farm animal welfare has become a major issue in European and other countries because of ethical reasons and new economic insights [1,2]. The poultry meat industry in particular faces a number of severe problems associated with modern methods of intensive rearing of poultry that elicit an increased interest in factors concerning welfare, and especially stress in birds [3,4]. According to Fraser [5], animal welfare is clearly a concept that can be studied scientifically, but the monitoring and quantification of poultry well-being is frequently related to physical (catching, handling) or psychological (fear, frustration) stress [6]. Blood analysis plays an important role in evaluating stress in birds, though there is little evidence of changes in blood parameters in poultry due to blood sampling *per se *[7]. The sampling techniques used to obtain these parameters are also invasive and stressful in themselves [8,9].

Physiological stress in birds has traditionally been assessed by measuring plasma corticosterone levels [10-12]. However, activities related to capture and blood sampling in birds can increase corticosterone levels and compromise meaningful stress assessment in research objectives [13,14]. Balcombe et al. [15], who have appraised eighty published studies to document the potential stress associated with routine laboratory procedures commonly performed on animals (handling and blood collection), indicate that the results of these studies demonstrate that animals responded with rapid, pronounced and significant elevations in stress-related responses. Significant changes in physiological parameters correlated with stress (e.g. serum or plasma concentrations of corticosterone, glucose, prolactin, heart rate, blood pressure and behaviour) were associated with both procedures in multiple species, including hens, ducks and geese, in the studies examined. Several studies [16-18] have identified manual catching and handling (as a component of transportation) as one of the most potent sources of stress and trauma in poultry. It is well documented (though predominantly in wild birds) that the duration of pre-sampling handling in particular influences hormonal levels, and corticosterone concentrations rise quickly [9,19-21]. However, only a few studies have directly tested whether glucocorticoid concentrations begin to increase in the first few minutes of capture in poultry, and the results have been incomplete, particularly regarding the possible effects of different handling techniques. Voslarova et al. [7] indicate that samples taken within 3 min of capture are likely to reflect unstressed concentrations of corticosterone in broilers. However, there is some confusion regarding the relative stressful and frightening properties of rough versus gentle capture and carrying [22]. On one hand, the adrenocortical responses of spent laying hens were consistently lower and less prolonged after gentle handling than were those of birds carried in the inverted position in a rough manner [23]. The results obtained by Kannan [24] also suggest that handling stress can be reduced in broilers by the use of upright handling. Conversely, Duncan [25] found no significant differences in the plasma corticosterone of broilers that were carried gently in an upright position and those which were inverted by the leg or exposed to deliberate rough treatment.

Since it is not yet clear whether it is possible to satisfactorily avoid handling-induced stress interference in poultry, more studies on the pattern of physiological response and detailed quantification of stress connected with the first few minutes of capture and pre-sampling handling in poultry are required. This study focused on detection of changes in the corticosterone level and concentrations of other selected biochemical parametes (albumin, glucose, cholesterol, lactate, triglycerides and total protein) in broilers handled in two different manners during blood sampling (involving catching, carrying, restraint, and blood collection itself) that lasted for various time periods within the interval 30-180 seconds. For monitoring, biochemical parameters were chosen whose concentration (in our opinion) may be influenced by the pre-sampling handling process, particularly when carried out in a rough manner. The study connects monitoring of the effect of various methods of pre-sampling handling in broilers and the duration of the whole process from catching to sampling itself.

## Methods

### Birds

The University of Veterinary and Pharmaceutical Sciences Brno Committee on Animal Care in Research gave approval of the experimental design through Protocol No. 02/2009.

Stress effects of pre-sampling handling were studied in a group of unsexed 42-day-old ROSS 308 broiler chickens. The broilers were obtained from a commercial broiler housing, where they were reared from the first day after hatching on deep litter (wood shavings) with controlled light and heat, hygiene, and feeding patterns corresponding to standard breeding requirements for meat hybrid poultry. Feed and water were provided ad libitum; the lighting regime was 18 hours of light and 6 hours of darkness. The ambient temperature was gradually decreased from 30 ± 1°C on Day 1 to 20 ± 1°C on the last day of fattening. On Day 35, the broilers (total number 180) were transferred to the experimental broiler housing at the University of Veterinary and Pharmaceutical Sciences Brno. The broilers were allowed to acclimate to their experimental environment for 7 days before experimentation began. The gender ratio of the broilers in the experiment was 50% to 50%. The effect of gender was not, however, monitored during the experiment, as our preliminary tests have shown this not to be statistically significant.

### Experimental Design

When the broilers were 42 days old, 144 birds (plus 36 "extra" birds) were taken at random for tests related to pre-sampling handling. Each of 12 pens contained 6 broilers randomly assigned to the following pre-sampling handling in a gentle manner and 6 broilers assigned to the pre-sampling handling in a rough manner. There were also three "extra" birds in each pen to minimize social stress caused by removal of experimental birds during tests. Handling involved catching, carrying into a separate room, restraint, and blood sampling itself. Birds were caught individually, with both hands, gently held in an upright position (gentle handling) or caught by the leg and held with a lack of care in an inverted manner (rough handling) and carried into a separate room, where they had no visual contact with other birds. The time between capture and transfer to the place where blood sampling took place (sampling table) did not exceed 10 seconds. Broilers placed on the table were restrained (gently or roughly respectively according to the group) by hand on their right side and blood samples (ca 3 ml) were drawn from the *vena basilica *with a needle 0.7 × 40 mm. The duration of restraint (including blood sampling) varied among six groups of 12 broilers each (i.e. one bird from one group per pen and handling manner): Group 30 s (30 seconds), Group 60 s (60 seconds), Group 90 s (90 seconds), Group 120 s (120 seconds), Group 150 s (150 seconds) and Group 180 s (180 seconds). To take into account possible diurnal changes in biochemical indices, blood sampling was performed progressively on one bird from each group according to handling duration (from Group 30 s to Group 180 s) and according to handling manner (gentle, rough), i.e. two birds (one gently handled, one roughly handled) were sampled at the same time for 30 s, two birds were sampled for 60 s etc. Each of the handling and sampling techniques used (rough and gentle) was performed by one separate pair of experimenters. This means, four (2 × 2) experimenters were used for sampling at the same time (i.e. one broiler was sampled by 2 experimenters in a rough manner and one broiler was sampled by 2 experimenters in a gentle manner at the same time). After the sampling, birds were removed from the experiment. This cycle of 6 × 2 sampling procedures was performed in 12 replications, meaning that a total of 144 samples were obtained. Because the effect of replication was not statistically significant, data were pooled before statistical analysis of treatment effects.

### Biochemical Examination

The blood samples were stabilized with heparin for biochemical examination (final heparin concentration in blood samples < 1%). The heparinized blood was centrifuged at 837 × g for 10 min and plasma samples were stored at -80°C in Eppendorf test tubes until analyses were performed (within 1 wk). Selected plasma biochemical indices, albumin, glucose, cholesterol, lactate, triglycerides and total protein were measured in a Cobas EMira biochemical analyzer using commercial test kits (Biovendor, Laboratorni Medicina AS, Modrice, Czech Republic). The plasma corticosterone concentration was measured using a commercial Corticosterone EIA Kit (Cayman Chemical, Ann Arbor, MI). Both assay procedures used for biochemical examination in the study are based on the principle of photometric determination.

### Statistics

The results were analyzed using the statistical package Unistat 5.1. (Unistat Ltd., GB). For all variables tested, normality was checked by means of a Shapiro-Wilk test (Zar, 1999). In the case of non-normal data (corticosterone, lactate), logarithmic transformations were used for analysis of variance, though actual mean values are presented in the tables. The data was subjected to ANOVA using the general linear model procedure with handling manner and handling duration as the main effects along with their interactions included in the model: *x_ijk _*= *μ *+ M*_i _*+ D*_j _*+ (MD)*_ij _*+ e*_ijk_*, where: *x_ijk _*= analyzed measurement, *μ *= overall mean, M*_i _*= effect of handling manner (gentle, rough), D*_j _*= effect of handling duration (30, 60, 90, 120, 150, 180 seconds), (MD)*_ij _*= effect of interaction, *ε_ijk _*= overall error (residual). When the effect was statistically significant, the Tukey-HSD test was performed as a post hoc test for pairwise comparisons. Spearman rank correlation coefficients were calculated between duration of handling (seconds) and monitored biochemical indices to assess correlations in the experiment.

## Results

The results of biochemical examinations of the two groups of broilers handled and sampled in two different manners (gentle or rough) for 30, 60, 90, 120, 150 and 180 seconds are given in Table 1.

**Table 1:** Selected biochemical indices of broilers (n = 144) handled in two different manners (gentle or rough) during blood sampling for varying time periods within the interval 30-180 s

	Indicator						
	
Effect	ALB (g/l)	CORT (ng/ml)	GLU (mmol/l)	CHOL (g/l)	LACT (mmol/l)	TG (mmol/l)	TP (g/l)
**Handling manner**							
Gentle	12.07^b^	0.82^b^	13.46^b^	2.65^b^	5.27^b^	0.75^b^	35.23^b^
Rough	12.63^a^	1.33^a^	13.95^a^	2.87^a^	7.93^a^	0.89^a^	36.72^a^
SEM	0.25	0.07	0.15	0.06	0.03	0.06	0.65
**Handling duration**							
30 s	12.56	0.72^b^	13.88	2.85	3.97^c^	0.85	36.35
60 s	12.24	0.75^b^	13.99	2.76	5.57^b^	0.92	36.05
90 s	12.4	0.73^b^	13.47	2.65	6.52^a,b^	0.73	35.43
120 s	11.8	0.87^b^	13.56	2.75	7.34^a,b^	0.78	35.01
150 s	12.31	1.67^a^	13.74	2.78	8.03^a^	0.82	36.09
180 s	12.76	1.70^a^	13.6	2.78	8.28^a^	0.81	36.82
SEM	0.44	0.11	0.26	0.11	0.04	0.11	1.14

**ANOVA**	**Significance (*P*)**						
H. manner (M)	0.027	0.009	0.001	<0.001	<0.001	0.028	0.022
H. duration (D)	N.S.	<0.001	N.S.	N.S.	<0.001	N.S.	N.S.
M × D	N.S.	N.S.	N.S.	N.S.	<0.001	N.S.	N.S.

ALB = albumin, CORT = corticosterone, GLU = glucose, CHOL = cholesterol, LACT = lactate, TG = triglycerides, TP = total protein

Means within the same effect and column lacking a common letter of superscript (^a, b, c^) differ significantly (*P *< 0.05).

It is evident from this table that pre-sampling handling carried out in a rough manner resulted in considerably higher plasma concentrations of all biochemical indices monitored when compared with gentle handling regardless of handling duration. We found a significant increase in plasma corticosterone (*P *= 0.009), glucose (*P *= 0.001), cholesterol (*P *< 0.001), lactate (*P *< 0.001), albumin (*P *= 0.027), triglycerides (*P *= 0.028), and total protein (*P *= 0.022). As is shown in the Table 1 concentration of plasma corticosterone after 180 s handling was considerably higher than concentrations after 30, 60, 90, and 120 s handling (*P *< 0.001, *P *= 0.004, *P *< 0.001, *P *= 0.026 respectively) regardless of handling technique. Similarly, corticosterone concentration after 150 s handling was also markedly higher than concentrations after 30, 60, 90, and 120 s handling (*P *< 0.001, *P *= 0.003, *P *< 0.001, *P *= 0.025 respectively) regardless of handling technique. At the same time, there were no significant differences between corticosterone levels after 150 s handling and 180 s handling, or between all groups of broilers handled for 30-120 seconds. Moreover, handling for 150 and 180 s carried out in a rough manner induced a significantly higher (*P *< 0.05) corticosterone level than handling for 30-90 s (Table 2). The results of two-way ANOVA revealed that an interaction (*P *< 0.001) between handling manner and handling duration was found in the case of lactate concentration (Table 1). As can be seen from this table, concentrations of plasma lactate increased with prolonged handling duration. Pre-sampling handling for 90, 120, 150, and 180 s resulted in a pronounced elevation of lactate concentration in comparison with 30 s handling (*P *< 0.001 in all cases respectively) regardless of handling manner. In addition, handling for 150 s and 180 s induced a significant increase in lactate level when compared with 60 s handling (*P *= 0.013, *P *= 0.012 respectively). As is shown in the table of interactions (Table 2), significant elevations in lactate concentration were found in broilers sampled in rough manner for 90, 120, 150, and 180 s when compared with gentle manner (*P *= 0.009, *P *= 0.001, *P *= 0.003, *P *= 0.003 respectively). Moreover, handling for 60, 90, 120, 150, and 180 s carried out in a rough manner induced a significantly higher lactate concentration than handling for 30 s (*P *= 0.001, *P *< 0.001, *P *< 0.001, *P *< 0.001, *P *< 0.001 respectively). No significant differences were found in albumin, glucose, cholesterol, triglycerides, and total protein levels among groups of broilers handled and sampled for various time periods in our study.

**Table 2:** Handling manner by handling duration interaction for lactate concentration of broilers (n = 144, means ± SEM).

Handling duration	Lactate (mmol/l)	
	
	Gentle manner	Rough manner
30 s	4.18 ± 0.32^b, x^	3.76 ± 0.19^c, x^
60 s	4.96 ± 0.35^a, b, x^	6.19 ± 0.22^b, x^
90 s	4.91 ± 0.28^a, b, y^	7.99 ± 0.76^a, b, x^
120 s	5.33 ± 0.31^a, b, y^	9.35 ± 0.83^a, b, x^
150 s	6.20 ± 0.48^a, y^	9.86 ± 0.63^a, b, x^
180 s	6.04 ± 0.52^a, b, y^	10.5 ± 0.81^a, x^

Means within the same handling manner and column lacking a common letter of superscript (^a, b, c^) differ significantly (*P *< 0.05).

Means within the same row lacking a common letter of superscript (^x, y^) differ significantly (*P *< 0.05).

Spearman rank correlation coefficients between length of handling (s) and monitored biochemical indices in broilers handled in a gentle and rough manner are presented in Table 3. The results indicate that length of pre-sampling handling (s) was significantly and positively correlated with the corticosterone plasma concentration (*P *< 0.001) and lactate level (*P *< 0.001) in broilers handled in both a gentle and rough manner.

**Table 3:** Spearman rank correlation coefficients between length of pre-sampling handling (s) and monitored biochemical indices in broilers handled in a gentle (n = 72) or rough manner (n = 72).

Indicator	Gentle handling		Rough handling	
		
	**Correl.coef**.	Signif. (*P*)	**Correl.coef**.	Signif. (*P*)
ALB (g/l)	0.032	N.S.	-0.007	N.S.
CORT (ng/ml)	0.503	< 0.001	0.385	< 0.001
GLU (mmol/l)	-0.118	N.S.	-0.095	N.S.
CHOL (g/l)	-0.175	N.S.	0.081	N.S.
LACT (mmol/l)	0.429	< 0.001	0.726	< 0.001
TG (mmol/l)	-0.036	N.S.	-0.071	N.S.
TP (gl/l)	-0.008	N.S.	0.046	N.S.

ALB = albumin, CORT = corticosterone, GLU = glucose, CHOL = cholesterol, LACT = lactate, TG = triglycerides, TP = total protein.

## Discussion

Evaluating welfare and stress that is assumed to correlate with the overall health status of an animal is difficult (particularly in birds), because sampling techniques used to obtain physiological parameters are also invasive and stressful in themselves [8,19]. Therefore, more studies on the pattern of detailed quantification of the pre-sampling stress response, particularly in poultry, are desirable. For this reason, this study focused on the temporal pattern of changes in selected biochemical stress indices in broilers following pre-sampling handling carried out in two different manners. The results of our study clearly showed that pre-sampling handling of broilers (i.e. catching, carrying, restraint, and collecting of blood itself) that was carried out in a rough manner had a much more pronounced impact on changes in the level of all biochemical parameters monitored in our experiment than pre-sampling handling carried out in a gentle manner. We found a highly significant increase in plasma concentrations of corticosterone, glucose, cholesterol, and lactate in broilers following rough pre-sampling handling in comparison with broilers handled and sampled in a gentle manner. First and foremost, the plasma level of corticosterone, which is traditionally used for assessment of physiological stress in birds [10,11,14] showed a marked elevation (from 0.82 to 1.33 ng/ml). This indicates a considerably higher stress load in roughly handled broilers in comparison with gently handled birds. In addition, rough handling also resulted in significantly elevated plasma levels of albumin, triglycerides and total protein in broilers when compared with gentle pre-sampling handling. Since an interaction between handling manner and handling duration was found in the case of lactate concentration only, we can conclude that the observed changes in the other biochemical indices were consistent across all time periods of pre-sampling handling monitored in our experiment. These results are in agreement with Knowles and Broom [23], who reported markedly lower and less prolonged adrenocortical responses of laying hens that were carried in an upright position and gently handled for 90 s than in birds that were handled and carried in the inverted position in a rough manner. Kannan [24] also suggests that handling stress can be reduced in broilers by the use of upright handling. In contrast, Duncan [25], who found elevated corticosterone in broilers in response to manual catching and restraint, mentioned that the stressor effects of such contact were so pronounced that inversion of the birds, or deliberate rough treatment, had no additional effect. He concluded that "it is handling *per se *which is stressful and not the rough manner in which it is carried out". Kettlewell and Mitchell [16] also have identified manual catching and handling as one of the most potent sources of stress and trauma in poultry.

The importance of collecting blood samples very quickly after capture when assessing baseline corticosterone concentrations has been emphasized by various authors, although predominantly in wild birds [9,19,21]. Research has shown that capture, handling and the blood sampling procedure itself can greatly influence corticosterone concentration in animals [7,13,14]. In our experiment, the most marked effect of the length of pre-sampling handling was expressed in a marked increase in corticosterone and lactate levels. The results of our analyses (as judged by corticosterone level) indicate that pre-sampling handling not exceeding 120 seconds, can be considered as a procedure that does not significantly affect baseline (unstressed) levels of biochemical indices in broilers. It also may be considered that corticosterone needs a minimum time of 120 s to show a significantly elevated concentrations due to pre-sampling handling in broilers. However, pre-sampling handling lasting for 150 seconds and more induced a considerable elevation in corticosterone concentration in broilers. The levels of corticosterone in birds exposed to handling for 150-180 s were more than two-fold those seen in broilers that were captured and sampled within 30-90 s. This indicates a pronounced stress response of birds following a sampling procedure of a length equal to or exceeding 150 seconds. The strong positive correlation between corticosterone level and duration of pre-sampling handling (Table 3) found in our study also supports this conclusion. In contrast, Voslarova et al. [7] reported that samples taken within 3 min of capture were likely to reflect unstressed concentrations of corticosterone in broilers. The results of Romero and Reed [9] indicate a high degree of confidence for the five avian species monitored in the study that samples collected in less than 2 min reflect unstressed (baseline) concentrations. Chloupek et al. [21] conclude that blood samples should be collected within 1.5 min to ensure that the levels of biochemical stress indices in common pheasants are not affected by stress induced by pre-sampling handling. But it is evident that pheasants, which are primarily wild animals, may show a markedly different stress response than domestic poultry, which has already been observed [26,27]. The results of our study showed lactate plasma concentrations to also increase markedly in response to the capture and handling process in broilers. This elevation was even more rapid than the corticosterone increase - as early as 60 seconds after capture. The lactate concentration was significantly higher than in broilers sampled within 30 s and rose further with a prolonged period of handling (from 3.97 to 8.28 mmol/l at 180 s). This rapid elevation in lactate level may be attributed to a considerable struggling activity of broilers during catching and the pre-sampling handling process. Similarly as with corticosterone concentrations, a strong positive correlation was found between plasma lactate and the duration of pre-sampling handling in our study (Table 3). These findings are in agreement with Bedanova et al. [12], who describe a very early onset of plasma lactate elevation in response to short-term stress in broilers, and also with Chloupek et al. [21], who found a significant positive correlation between lactate concentration and duration of pre-sampling handling in common pheasants. Similarly, Ali et al. [28], who studied the influence of pre-slaughter stress (in connection with handling and shackling process) in poultry, found increasing lactate levels with increasing struggling activity of broilers. In contrast, Voslarova et al. [7] reported a significant increase in lactate plasma concentrations only in broilers handled and sampled for more than 3 min.

It is well known that stress stimulates the body to release glucose and other energetic metabolites into the blood stream in physiologic preparation for the "fight or flight" response. When monitoring the stress effect of pre-sampling handling in our study, the acute mobilisation of energy sources (glucose, total protein, albumin, triglycerides, cholesterol) was expected as a result of the stress load on the broilers during the pre-sampling handling process. This did not, however, occur during the monitored time period, which may be attributed to the onset of the stress response for these biochemical indices occurring after a period longer than 180 seconds. Similarly, Voslarova et al. [7] also reported no changes in glucose and cholesterol levels within 6 min of pre-sampling handling in broilers. In contrast, Bedanova et al. [12] describe a significant elevation in the glucose level after 60 seconds of pre-slaughter handling in broilers. Chloupek et al. [21] found a significant increase in the glucose level in response to 3 min of pre-sampling handling in pheasants, but no changes in the cholesterol level within 6 min of handling. Balcombe et al. [15] describe an early (within 2 min) elevation of plasma glucose and total protein in rats in response to handling and sampling. Concentrations of albumin, glucose, cholesterol, triglycerides, and total protein found in our study did not show any correlation pattern in connection with duration of pre-sampling handling in broilers, which also may support a latter than 180 s onset of stress response in these biochemical indices.

## Conclusions

In conclusion, the results presented indicate that pre-sampling procedures may be a considerably stressful event for broilers, particularly when carried out with a lack of care and exceeding 120 seconds. This indicates that blood samples from broilers should be collected within 2 min of capture in order to ensure that the plasma levels of corticosterone and lactate in particular are not influenced by the stress effects of handling, or immobilization stress, inevitable when blood samples are taken.

## Competing interests

The authors declare that they have no competing interests.

## Authors' contributions

PCh designed and coordinated the study and was involved in collecting the blood, drafting the manuscript and revising it critically. IB carried out the statistical analysis and wrote the manuscript. JCh contributed to conception and design and collected the blood samples. VV contributed to the design and planning, made substantial contributions in revising the manuscript critically and gave final approval of the version to be published. All authors read and approved the final manuscript.
